# Adverse Neighborhood Environments and Health Behaviors among Older Americans

**DOI:** 10.1093/geroni/igab046.3661

**Published:** 2021-12-17

**Authors:** Yeon Jin Choi

**Affiliations:** University of Southern California, Los Angeles, California, United States

## Abstract

Maintaining healthy lifestyle, including healthy diet and physical activity, in adverse neighborhood environments may be more difficult for older adults because of changes linked to aging, which make them more vulnerable to their environments. This study aims to investigate the association of neighborhood disorder with diet quality and physical activity in a national sample of older Americans. For this study, we used data from the Health and Retirement Study. Neighborhood disorders include vandalism, boarded houses, abandoned cars, demolished houses, trash, litter, or junk, poorly kept communal areas, homeless people, prostitution, winos or junkies, and drug use or drug dealing near residents’ housing unit (range: 0-11). Diet quality and physical activity were assessed using the Healthy Eating Index 2015 (HEI-2015; range:0-100) and the metabolic (MET) equivalent activity points (range: 0-31 in this sample). Ordinary least squares regression models were estimated to examine an association between neighborhood disorder, diet quality, and physical activity. Neighborhood disorder was associated with poor diet and physical inactivity. For one additional negative neighborhood feature, HEI-2015 scores and MET-equivalent activity points decreased by 0.55 (95% CI: -1.09. -0.01) and 0.69 (95% CI: -1.05, -0.33). Findings of this study suggest that older adults living in adverse neighborhoods are at a greater risk of poor diet and physical inactivity, which are important risk factors for poor health and chronic diseases. Promoting neighborhood environments and perceived neighborhood safety would increase access to health food, encourage healthy diet and physical activity, and support healthy aging.

